# Clinical characteristics and microperimetry of patients with myopic maculopathy referred to vision rehabilitation compared to age related macular degeneration

**DOI:** 10.1371/journal.pone.0356824

**Published:** 2026-09-15

**Authors:** Cody Lo, Mary Lou Jackson

**Affiliations:** 1 Department of Ophthalmology and Visual Sciences, Faculty of Medicine, University of British Columbia, Vancouver, British Columbia, Canada; 2 SFU Stephens School of Medicine, Simon Fraser University, Surrey, British Columbia, Canada; University of South Florida, UNITED STATES OF AMERICA

## Abstract

Myopic maculopathy (MM) is a progressive degenerative change of the retina and choroid associated with high myopia leading to central vision loss. The burden of MM is anticipated to increase due to the rising prevalence of myopia globally. Patients with MM are often referred for vision rehabilitation (VR) to help adapt to visual impairment. VR aims to allow patients to continue performing tasks and remain safe despite vision loss, typically through training with optical devices, assistive technology, or new strategies. This study compares the presenting clinical characteristics and VR process of patients with MM to patients with age-related macular degeneration (AMD), the latter being the most common diagnosis of patients referred for VR. This retrospective chart review included 32 consecutive patients with MM and 32 randomly selected patients with AMD referred for VR. Only patients with visual acuity in either eye better than 20/200 were included to represent the early stage of both disease processes. Available microperimetry data were collected for all eyes with a visual acuity better than 20/200 and graded for the presence of scotomas by two independent reviewers. Patients with MM were significantly younger (p < 0.0001) than those with AMD (mean age 68.0 vs 81.6 years respectively). Reported priority goals for vision rehabilitation showed a significant trend: patients with MM and moderate acuity prioritized daily living tasks more frequently (67%, p = 0.0045) than patients with AMD. At good acuity (>20/50), dense paracentral scotomas trended towards being more prevalent amongst patients with MM compared to moderate acuity (between 20/50 and 20/200) where central scotomas were more prevalent amongst patients with AMD. Patients with early MM have unique characteristics at presentation for VR compared to patients with AMD. It is important for providers to be aware of the visual challenges faced by younger patients with paracentral scotomas, which may warrant VR referral even when visual acuity is good.

## Introduction

Myopic maculopathy (MM) is a progressive degenerative change of the retina and choroid associated with high myopia that leads to central vision loss [1]. MM is characterized by diffuse and patchy chorioretinal atrophy, lacquer cracks, choroidal neovascularization and macular atrophy [2]. Myopia affects an estimated 2.6 billion people worldwide with a prevalence of over 50% in many countries throughout Asia [3–5]. The current prevalence of MM is estimated to be 3% worldwide [6] but varies with some of the highest rates reported in countries such as Singapore (15%) [7]. Patients with MM can experience loss of vision that can impact many areas of their function such as their ability to drive, ambulate safely, or complete tasks related to their employment [8].

Vision rehabilitation (VR) assists patients with visual impairment in achieving their goals and maintaining safety and quality of life [9,10]. Interventions in VR shown to have benefit vary widely from optical or electronic magnifying devices, standardized training to use devices, text-to-audio technology, and patient support groups [11]. Two recent Cochrane reviews evaluating studies of VR interventions point to the value of future research identifying patient characteristics which can differentiate types of patients referred to VR and may predict performance with different rehabilitation interventions.

Microperimetry is a technique that images the retina with a scanning laser ophthalmoscope (SLO) or camera during perimetry testing of the central visual field [12, 13]. This achieves higher accuracy compared to standard perimetry methods for patients with poor fixation due to disease affecting central vision, such as maculopathies.[12,14] Microperimetry is increasingly used in VR research to understand central field defects and fixation characteristics [15].

To date, there has been little investigation of VR for patients with early MM. We are particularly interested in those with early-stage disease as these patients often have good acuity and may not be referred to VR despite experiencing visual challenges. This study retrospectively reviewed patients referred to a tertiary VR clinic with a diagnosis of early MM compared to patients referred with a diagnosis of AMD, to understand the presenting characteristics of patients with MM compared to other patients seeking VR.

## Materials and Methods

### Myopic maculopathy cohort

This retrospective chart review was conducted at the Vision Rehabilitation Clinic of the VGH/UBC Eye Care Centre in Vancouver, Canada which is a tertiary centre serving British Columbia, Canada. Inclusion criteria included a referring diagnosis of MM, age greater than 18 years and consultation at the VR department from March 25, 2020 to March 25, 2021. All participants were assessed by an ophthalmologist subspecializing in vision rehabilitation (M.L.J.) who personally conducted the history asking about their visual challenges and priority goals for vision rehabilitation. The examiner was able to clarify their questions based on the patient’s comprehension. Additionally, participants needed a best corrected visual acuity (BCVA) better than 20/200 in either eye. Cases were excluded if they had any other ocular comorbidity which could also require visual rehabilitation such as glaucoma or retinal dystrophies. Additional cases referred with a diagnosis of “myopia” were reviewed by one author (M.L.J.) for features diagnostic of myopic maculopathy as outlined by the META-PM classification [16]. A consecutive cohort of participants meeting these criteria was included in this study. The research study was approved by the University of British Columbia Research Ethics Board (H20-03530 and H22-00196) and fully complied with the tenets of the Declaration of Helsinki. Given the study’s retrospective nature, the ethics committee did not require informed consent from participants. All data were fully anonymized during data collection. The file linking the study ID to identifying patient information was kept separate from the study data and was accessible only to one author (M.L.J.), who had a clinical relationship with the participants. Participants were excluded if they indicated on their chart that they did not wish to participate in retrospective research. Charts were accessed for data collection from January 01, 2021 to June 30, 2021. A subset of charts were reviewed during data analysis from April 01, 2022 to December 31, 2022.

### Age-related macular degeneration cohort

AMD is a maculopathy like MM and is also the most common referring diagnosis to both the study site and in a large study of 28 VR centres in the US. [17]. A comparator cohort was created of patients referred with a diagnosis of AMD using the same inclusion and exclusion criteria as the MM cohort. The control cohort was randomly selected from patients seen in the clinic during the same 12-month period as the MM cohort and matched 1:1 to the MM cohort by visual acuity at referral.

### Microperimetry testing

Microperimetry testing was obtained using the Macular Integrity Assessment system (MAIA; CenterVue, Padova, Italy). Monocular fields from each eye that saw better than 20/200 were reviewed by two independent reviewers (C.L. and M.L.J.) for sufficient quality (i.e., absence of other retinal pathology) and then categorized into one of 3 broad patterns of central field loss: 1) no dense scotoma: 2) paracentral dense scotoma; or 3) central dense scotoma. A central scotoma was defined as: 1) any scotoma that involved the test point closest to the point of foveal fixation on the SLO image; or 2) a patient who developed a non-foveal preferred retinal locus (PRL) and the SLO shows the centre of the fovea within the scotoma. A paracentral scotoma was defined as a scotoma that did not involve the test point closest to the point of foveal fixation on the SLO image.

### Data extraction

Clinical notes from the subjects’ initial referral and VR consultation were reviewed for clinical characteristics. Basic demographic information such as age, gender, past ocular and surgical history was obtained from the referral note. The priority goal for rehabilitation reported by the patient during VR consultation was determined by one author (M.L.J.) based on the documented narrative in the clinical note. The goals were classified into five categories based on the multidisciplinary model of comprehensive VR outlined in the American Academy of Ophthalmology Preferred Practice Pattern (AAO PPP): reading, daily living activities, safety, continued participation in activities despite vision loss, and psychosocial well-being [8].

### Data analysis

Quantitative data were summarized using Microsoft Excel for Mac (Version 16.40, Redmond, WA) to calculate descriptive statistics. Non-paired Student’s t-test was used to compare continuous variables. Fisher’s exact and chi-square test were used for binary and multiple non-continuous variables respectively, given the small sample sizes. Statistical tests were completed using IBM SPSS (Version 28, New York, United States). An initial p-value threshold of 0.05 was considered statistically significant, followed by application of the Bonferroni correction for multiple comparisons. The Bonferroni correction resulted in the loss of significance for two results in the analysis: 1) higher rates of reading as a priority goals amongst patients with AMD at moderate visual acuities (P = 0.03) and 2) higher rates of no dense scotomas in patients with MM at moderate acuities (P = 0.05).

To compare the clinical characteristics between the MM and AMD cohorts, each cohort was stratified by best-corrected visual acuity in the better-seeing eye, a surrogate for disease severity. The cut-off of “good” acuity was set at 20/50 or better as it is the threshold for driving in many jurisdictions. “Moderate” acuity was between 20/50–20/200 with the cut off being chosen as the threshold for “legal blindness”.

## Results

### Clinical characteristics at referral

Over 900 new patients were seen by the VR service over the study period. A total of 32 patients referred to VR with a primary diagnosis of MM met the inclusion criteria. The majority were referred with a known diagnosis of myopic maculopathy from an ophthalmologist. Two cases were initially referred with a diagnosis of “myopia” but were never documented to have maculopathy until assessment by the VR service. Over 300 patients with AMD were seen over the study period. This allowed for a roughly 1:10 sampling of patients with AMD seen through the 1-year study period to create the control cohort. The distribution of subjects by visual acuity at referral did not differ significantly between the MM and AMD cohorts. Patients with MM were 68.0 years old on average (SD 15 years) at referral and significantly younger (p < 0.0001) than those with AMD, who had a mean age of 81.6 years (SD 6.4 years). A total of 18 participants (28%) were male, with no significant difference between the MM and AMD cohorts.

### Priority goals of vision rehabilitation

Qualitative analysis of the narratives provided by patients during VR consultation revealed that their priority goals in vision rehabilitation could be classified into 5 goals set out by the AAO PPP for vision rehabilitation. Reading and the ability to perform daily living activities (i.e., tasks at home, using a computer) were the two most common priorities amongst both patients with MM and AMD across both levels of presenting visual acuity (Table 1). The daily living activities as described in the AAO Preferred Practice Pattern (PPP) guideline most mentioned by patients with MM were using a telephone, cell phone, tablet, or computer and enjoying leisure activities.

**Table 1:** Priority goals of vision rehabilitation reported patients with early myopic maculopathy compared to age-related macular degeneration and possible interventions.

Priority goal (n, %)	Good acuity (20/50 or better)			Moderate acuity (between 20/50 and 20/200)		
	MM (n = 14 patients)	AMD (n = 18 patients)	P value^1^	MM (n = 18 patients)	AMD (n = 14 patients)	P value^1^
Reading	6, 43%	12, 67%	0.28	5, 28%	10, 71%	0.03
Daily living activities^2^	4, 29%	5, 28%	0.36	12, 67%	2, 14%	0.0045**
Safety	2, 14%	0, 0%	0.18	1, 6%	2, 14%	0.57
Continued participation in activities despite vision loss	1, 7%	0, 0%	0.44	0, 0%	0, 0%	1.00
Psychosocial well-being	1, 7%	1, 6%	1.00	0, 0%	0, 0%	1.00

**indicates statistical significance

1. P value of 0.005 was considered significant after Bonferroni correction

2. Daily living activities as described in the AAO Preferred Practice Pattern (PPP) Guideline include: using a telephone, cell phone, tablet, or computer; paying bills, counting money, and managing finances; shopping and financial management; preparing and eating meals; seeing faces; watching TV, a movie, or a theater performance; managing personal self-care; managing glare and lighting; enjoying leisure activities

### Microperimetry

**Fig 1:**
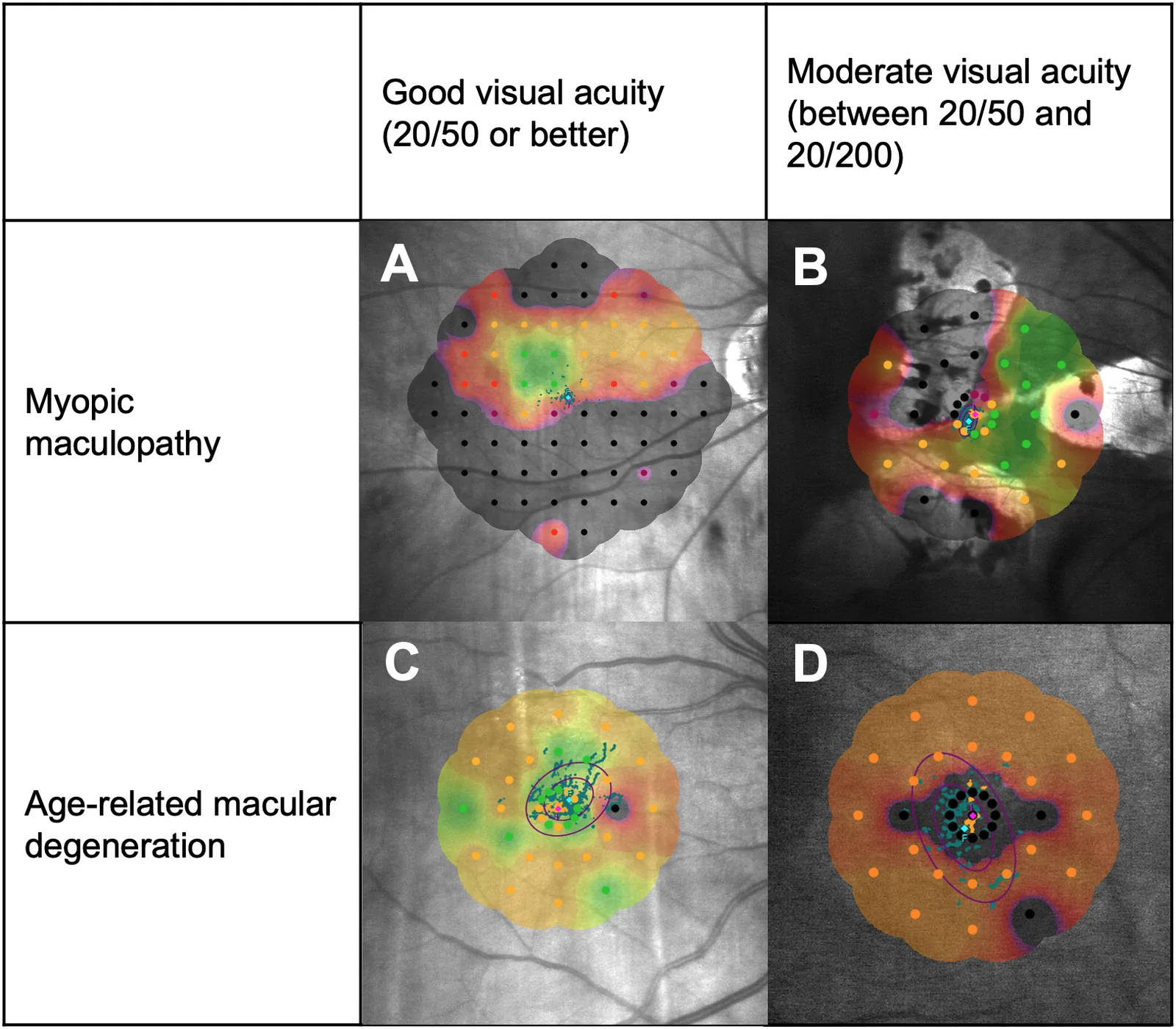
Microperimetry tests of patients with myopic maculopathy (A, B) and age-related macular degeneration (C, D) at both good (20/50 or better) and moderate (between 20/50 and 20/200) visual acuities.

Of the 64 patients included in this study, 42 (65%) had microperimetry of both eyes, 15 (23%) had microperimetry of 1 eye, and 7 (11%) did not have microperimetry of either eye. Generally, there are significantly fewer patients (P = 0.03) with MM who undergo microperimetry for both eyes (34%) than patients with AMD (78%), due to technical challenges related to anatomic distortions of the myopic fundus. Distortions due to posterior staphyloma or high axial length can make fundus tracking difficult but patients can be coached to maintain steady fixation to mitigate this. After assessing for image quality, there were 91 microperimetry results included in the study among all patients. Amongst 91 microperimetry images that could be analyzed, 27 patients had both eyes included. Two separate analyses were done to account for inter-eye correlation in the same patient. The kappa value for classifying scotomas types on microperimetry was 0.883, indicating very strong agreement between graders. Fig 1 illustrates representative microperimetry images from each visual acuity group and disease cohort. Dense paracentral scotomas can be present in eyes with MM despite good visual acuity. (Fig 1A). For those with moderate acuity loss, there was a trend towards patients MM having paracentral scotomas encompassing most of the macula (Fig 1B). In patients with AMD there is typically early localized central involvement (Fig 1C) that enlarges as visual acuity worsens (Fig 1D). Table 2 describes the quantitative classifications of the microperimetry data for one randomly selected eye per subject in the cohort to account for any inter-eye correlations. S1 Table is provided as a sensitivity analysis including all eyes in the cohort with microperimetry available and show similar trends. At good and moderate acuities, there were trends towards different types of scotomas in patients with MM compared with those with AMD. At good acuity, dense paracentral scotomas were more prevalent amongst patients with MM compared to patients with AMD (Table 2) but this trend was not statistically significant (P value = 0.19). At moderate acuities, there was a trend towards no dense scotomas being more common in patients with MM (P value = 0.05).

**Table 2:** Microperimetry characteristics of patients referred to vision rehabilitation with myopic maculopathy (MM, n = 31 eyes) and age-related macular degeneration (AMD, n = 32 eyes) by presenting acuity [1].

Type of scotoma (n, %)	Good acuity (20/50 or better)			Moderate acuity (between 20/50 and 20/200)		
	MM (n = 10 eyes)	AMD (n = 15 eyes)	P value	MM (n = 21 eyes)	AMD (n = 17 eyes)	P value^2^
No dense scotoma	3, 30%	7, 47%	0.68	13, 62%	5, 29%	0.05
Paracentral scotomas	5, 50%	3, 20%	0.19	12, 57%	13, 76%	0.79
Central scotomas	2, 20%	5, 33%	0.66	6, 29%	11, 65%	0.15

1. For patients where microperimetry was available for both eyes, one eye was randomly selected to be included in analysis to account for any inter-eye correlations

2. P value of 0.008 was considered significant after Bonferroni correction

## Discussion

This retrospective chart review describes the clinical characteristics and microperimetry images at initial referral to VR for patients with early MM compared with those with AMD. Patients with MM referred to VR while still having good visual acuity are typically younger, have a trend towards more dense paracentral scotomas despite retained visual acuity. Patients with early MM can have challenges related to paracentral vision loss such as difficulty finding their cursor on their computer monitor, finding objects in a store, or using their smartphone. Interestingly aside from daily living activities, we did not see any difference in priority goals between patients with MM and AMD. It is possible this is due to only the most important goal, as determined from their consult visit, was recorded. It is possible that if a ranking system was used there may be differences in the 2^nd^ or 3^rd^ most important goals for example. We found that patients with MM were significantly younger than those with AMD which is anticipated given the disease demographics. However, this is still important for practitioners to recognize as patients with MM having visual challenges may still be in their working years and low vision can impact employment or ability to generate an income. For younger patients, paracentral scotomas can hinder computer use or reading leading to premature career exit, highlighting the need for early VR referral.

Few studies have reviewed microperimetry data in the setting of myopic maculopathy (Fig 1 and Table 2) [18,19]. This study found a greater proportion of patients with MM at good acuities to have dense paracentral scotomas compared to patients with AMD at moderate acuity who more often had central scotomas. The contrasting central field characteristics in each group could explain the greater reports of reading difficulties in the AMD cohort and the greater reports of difficulties with tasks associated with paracentral field loss (i.e., losing the computer cursor or misplacing items) in the MM cohort. Density of a scotoma may be relevant to visual functioning as a small but dense scotoma in MM might be more functionally disruptive than a larger, relative scotoma in an AMD patient. There is little empiric evidence demonstrating the impact on visual functioning on paracentral scotomas close to fixation. Most previous studies are focused on scotomas from glaucoma or strokes. However particularly relating to detection of hazards while driving, there are multiple studies that suggest impaired reaction time with scotomas adjacent to fixation [20,21]. Younger age may have also contributed to the difference in priority goals, as younger patients are more likely to be working.

Previous studies characterizing the visual complaints of 819 new VR patients in the US found that the most common complaints were reading (66.4%), driving (27.8%), and in-home activities (15.1%) with 77% of those with atrophic AMD reporting reading as their primary difficulty [22]. Our results for the priority goals of patients with AMD are in keeping with this study; however, our study adds to the existing literature by outlining the goals of patients with early MM. The prior study did not specifically consider patients with MM. Recognizing the unique needs of this cohort may inform effective rehabilitation planning for patients with MM.

There are several limitations of this study. Data collection was retrospective and although all microperimetry images were obtained by a single operator, there was no standardized protocol for their acquisition. Therefore, it was not possible to compare common quantitative metrics from microperimetry between all studies such as mean sensitivity (MS) in decibels (dB), scotoma area (number of affected points), or bivariate contour ellipse area (BCEA) for fixation stability. Additionally, data collection took place between 2020 and 2021 during the COVID-19 pandemic. At the study location, routine outpatient visits were suspended from March 2020 with a gradual return to full volumes starting mid-May 2020. While British Columbia experienced a relatively short institutional and regional lockdown restriction period compared to other Canadian jurisdictions, the pandemic context and associated social disruptions may have influenced the priority goals reported by patients during their consultations.

Patient ethnicity was not recorded in the medical charts and could not be obtained retrospectively under the parameters of our current Research Ethics Board (REB) approval. Given the well-established ethnicity gradient in the prevalence and severity of high myopia and myopic maculopathy, particularly its higher prevalence in Asian populations, the lack of demographic breakdown regarding ethnicity limits the generalizability of our findings to broader global populations. Age may confound our findings related to reported priority goals and should be controlled for in future research. There are also other possible confounders such as contrast sensitivity which can reduce visual functioning independent of visual acuity which may have impacted the results. The available sample size was too small to allow for age-matched recruitment and maybe underpowered for the trends that did not show statistical significance here. This study was conducted at a single tertiary academic centre, and this patient population may not be representative of other settings [23,24]. A potential limitation of this study is referral bias, as the study cohort may not be representative of the general population. There is no standardized method for describing microperimetry or categorizing scotoma patterns from microperimetry examinations so our methods may have introduced bias despite the interpretations being agreed on by two independent reviewers. However, future studies could use standardized perimetry protocols which would allow for comparison of quantitative metrics such as MS across studies. Quantitative comparison of microperimetry deficits and their correlation with retinal structural damage and vision rehabilitation outcomes would be a valuable topic for future research.

As the incidence of myopia and myopic maculopathy increases globally [3,6,7], it is important to recognize that patients with early MM have unique challenges and goals when referred for VR. Given their younger age, they are often still working and have rehabilitation goals specific to their vocation. This study found that patients with MM often report difficulty using vision for daily living tasks, even when visual acuity remains good. This may be related to increased rates of paracentral scotomas identified on microperimetry in this cohort. Some practical interventions for those providing VR to these patients include tailoring the approach based on etiology. For patients with AMD and central scotomas interventions focus on options for magnification. Whereas patients with MM and paracentral scotoma may benefit from a focus on digital accessibility (i.e., text-to-speech), lighting optimization, and strategies to compensate for objects, such as cursors, disappearing into paracentral scotomas. Patients with MM may have good central vision for reading but “navigation” within a page is impaired. Our results suggest that patients with MM often have difficulties with daily tasks due to paracentral scotomas and may benefit from early referral to VR.

## Supporting information

S1 Table Microperimetry characteristics of all eyes of patients referred to vision rehabilitation with myopic maculopathy (MM, n = 43 eyes) and age-related macular degeneration (AMD, n = 48 eyes) by presenting acuity. (DOCX)
