# Supplementary material for: Clinical characteristics and microperimetry of patients with myopic maculopathy referred to vision rehabilitation compared to age related macular degeneration
Source: PLoS One. 2026 Sep 15;21(9):e0356824. doi: 10.1371/journal.pone.0356824 (PMC13577545; doi:10.1371/journal.pone.0356824)
Supplement: Supplementary file 1 [file pone.0356824.s001.docx]

**Supporting Information**

**S1 Table. Microperimetry characteristics of all eyes of patients referred to vision rehabilitation with myopic maculopathy (MM, n=43 eyes) and age-related macular degeneration (AMD, n=48 eyes) by presenting acuity.^1^**

| Type of scotoma (n, %) | Good acuity  (20/50 or better) | | | Moderate acuity (between 20/50 and 20/200) | | |
| --- | --- | --- | --- | --- | --- | --- |
|  | MM (n= 12 eyes) | AMD (n= 19 eyes) | P value | MM (n= 31 eyes) | AMD (n= 29 eyes) | P value^2^ |
| No dense scotoma | 3, 25% | 9, 47% | 0.27 | 13, 42% | 5, 17% | 0.05 |
| Paracentral scotomas | 7, 58% | 5, 26% | 0.13 | 12, 39% | 13, 49% | 0.79 |
| Central scotomas | 2, 17% | 5, 26% | 0.68 | 6, 19% | 11, 38% | 0.15 |

1. Both eyes were included for patients where microperimetry was available

2. P value of 0.008 was considered significant after Bonferroni correction
